# Observations on the Use of the Obstetrical Extractor

**Published:** 1851-05

**Authors:** Jno. Evans

**Affiliations:** Prof. of Obstetrics, &c., in Rush Medical College


### ARTICLE VI.

*Observations on the use of the Obstetrical Extractor.* By JNO. EVANS, M. D., Prof. of Obstetrics, &c., in Rush Medical College.

It is now over twelve months since I commenced the use of this instrument, and just one year since I gave an account of the invention to the public, during which time I have applied it twelve times; twice in cases which occurred in my own practice, and in ten cases where I had been called in attendance with other practitioners. Five of these cases were reported in the transactions of the American Medical Association for 1850; to which I will now add an account of its use in the cases that have occurred subsequently to that report:

CASE 1st. An Irish woman in the care of a midwife had been in labor twenty-four hours with her first child, when I was called. I found the head engaged in the superior strait; but not descended so far as to press upon the pericranium. It was a left occipito-anterior presentation. The uterine contractions were very strong, but, for the three hours that I attended, did not the least advance the child. I placed the patient in the usual position for applying the forceps, and proceeded to apply the extractor after the manner already described. This was done with such entire facility that I at once became satisfied of the utility of the invention; my only fear having been in reference to difficulties in its application. The labor was speedily and happily terminated without apparently increasing the patient's suffering over that that is natural, and with entire safety to both mother and child.

CASE 2d. Miss ——— had been in labor with her first child twenty hours, in care of a root doctor, when I was called in counsel with Prof. Herrick, in the case. We found the os uteri well dilated, the contractions forcible, and the head engaged in the superior strait, with the occiput forward and to the left. No advancement had been made for several hours, when it was determined to apply the extractor. The obstruction arose from the large size of the child. The application was made as before, and delivery as speedily and safely effected.

CASE 3d. Mrs. B. had been in labor for ten hours; the pains were beginning to grow weaker, and the advancement of the child, which presented naturally, had ceased for two hours. It was simply a case of tedious labor from inefficient uterine contractions, and I determined to apply the extractor, if possible, without the patient's knowledge of it. Of this intention I apprised her mother, who furnished me the means of preparing the instrument in an adjoining room. As the patient lay in the usual position for delivery, on the left side, I applied the extractor without causing so much pain as to lead her to suspect that I was using an instrument; though thinking I made an unusual examination, she inquired if anything was wrong. The delivery was effected speedily, and with safety to both mother and child. In this case, the band slipped over the chin of the child, after which I put the network below the band to prevent the like accident from occurring in future.

CASE 4th. An Irish woman who had been in labor twelve hours, in care of Prof. N. S. Davis, was much exhausted from long continued and violent expulsive efforts. The head was engaged in the brim of the pelvis, but had not descended into the concavity of the sacrum, and had made no advancement for several hours.

In attempting to apply the extractor, I found some difficulty in approximating the handles closely enough to receive the slide, but found that it was occasioned by the band not having been carried far enough up, as was proved by the instrument's coming away upon the application of force. The reason it was not carried high enough at first was a deception as to the actual position of the head. occasioned by great tumefaction of the scalp. A subsequent trial succeeded perfectly, and the delivery was accomplished without farther difficulty and with entire safety to both mother and child.

Case 5th. Mrs. A. had been in labor with her fourth child fifteen hours, in the care of Dr. J. S. King, of Chicago. I found the head entirely above the superior strait of the pelvis. Dr. K. observed that, although the soft parts seemed to be well relaxed and the contractions strong, there was no tendency during the pains to advancement of the head. The pains had been strong, without altering the position of the child, for about eight hours ; but the intervals between pains were now longer, and the contractions less forcible. The os uteri was dilated to two inches in diameter ; the occiput presented nothing upon the symphysis pubis. The Extractor was applied without any difficulty, and the delivery effected safely to mother and child within fifteen minutes from the time the application was commenced. The head of the child scarcely presented a trace of the instrument in any case in which it has been used. The only mark it has yet made has been slight redness and temporary depression of the skin, where the band and straps were applied.

In this case, the mother did well, but the child died in about twelve hours of morbus ceruleus,

Case 6th. Mrs. ———, in July last, had been in labor thirty-six hours, first under the care of a homœopathist, and subsequently of Dr. P. McGirr, of Chicago, when I was called. The uterine contractions were very strong and recurred quite frequently, but there had been no advancement of the child for several hours. The head presented in the first position, the left occipito-anterior of the vertex. It had descended so as to bring the forehead into the sacral excavation. It receded during the uterine relaxations, and came to the same position again during the pains. The homœopath had assured them twelve hours previously that the child would be born with a few more pains ; but the attendants assured us that she had been in equally hard labor for the whole of this period. The woman was much exhausted, and I applied the extractor with but little difficulty; but upon making traction upon the *straps** the *band* gave way, owing to a slight rent that had been made on a former occasion not having been repaired. I was obliged to deliver with the forceps. The mother and child both did well.

CASE 7th. Mrs. W——, an Irish woman, in care of Dr. Simple, of Chicago, had been three days in labor with her first child. Presentation, the left occipito-anterior of the vertex; head descended as in the preceding case; os tincæ quite fully dilated, but no advancement by the uterine contractions for several hours, notwithstanding they were of the most violent character. The first attempt to apply the extractor failed, because I did not carry the band far enough into the uterus, to place it around the face, and it slipped off. A second attempt was completely successful, and the child was speedily and safely delivered. Mother and child both did well.

CASE 8th. Mrs. ———, whose pelvis is contracted, and whom I had, in consultation with Prof. Davis, delivered with the forceps, of a still-born child a year previously, was taken in labor at the full term, in November last. When I arrived the child's head had descended into the pelvic excavation, and was so firmly impacted that it was with much difficulty that I passed the fingers of the extractor around it. I observed to Prof. Davis, in whose practice the case occurred, that one having less interest in the extractor might have failed of its application. When applied, it required much force for a considerable time, as it had done on the previous occasion with the forceps, to effect delivery. The child was still born. This is the only case where the child has been born dead in which the extractor has been used, excepting one where it was known to be dead before its application.

*For description of the extractor see N. W. Med. & Surg. Jour. for May, 1850, or Transactions of American Medical Association for 1850.

Case 9th. Mrs. L—— had been in labor twelve hours, in care of Prof. Herrick, February 4th, when I was called. Right occipito-posterior presentation of the vertex, and notwithstanding there had been violent labor for several hours there was no advancement of the child. We each made an unsuccessful effort to bring down the vertex into the concavity of the sacrum with the vectis. I then applied the extractor, introducing it by the side of the head of the child and carrying one finger over its face, under the arch of the pubes, and found it impossible to get it further. I therefore passed the other around to meet it at that point where the slide was placed upon the handles; delivery was then effected without further difficulty. A contusion of one of the child's cheeks, made by the finger of the extractor, was the only disagreeable incident in the use of the instrument. Mother and child both did well.

Case 10th. Mrs. W—— in labor, in care of Mrs. Kelly, midwife, for twelve hours; occipito-anterior presentation of the vertex; was, from long continued labor, much exhausted. The pains were quite inefficient and no advancement had been made for several hours. In presence of Mr. Wright, a student, I applied the extractor, and speedily and safely delivered. Mother and child both did well.

Case 11th. March 12. Mrs. S——, aged thirty-three, with her first child, had been in labor twenty-four hours. The presentation occipito-anterior of the vertex; os tincæ soft, dilated to the size of a dollar and inclined to the promontory of the sacrum; violent contractions for several hours, with strong bearing down efforts failed to urge the head into the superior straight of the pelvis. I determined to apply the extractor but found it difficult to pass a finger of the instrument between the occiput of the child and the pubes of the mother. I took the instrument away, readjusted it, and depressing the handles, without difficulty passed it at once over the occiput and carried its fingers in opposite directions until they met on the face, in the concavity of the sacrum, where they were fixed by the slide upon the handles. With strong extractive force applied during each contraction of the uterus, the child advanced but slowly. But delivery was effected in about one hour and a half, the patient lying in bed on the left side during the time. The cord was around the neck and the child asphyxiated. After some time it was resuscitated, and both the mother and child did well.

CASE 12th. Mrs. T——, in care of Dr. S., of Chicago, had been in labor twelve hours when I was called. On examination, I found the umbilical cord cold and pulseless, and one hand of the child lying in the vagina. The vertex presented at the superior straight, the head resting in the right illiac fossa. I passed the extractor up, between the descending arm and head of the child, separated the handles and carried the fingers of the instrument in opposite directions until they met on the opposite side of the head, and fixed them by the slide without difficulty. The head was then readily drawn down and delivery soon effected. The woman did well. Had the extractor been applied in this case before the death of the child, I have but little doubt it might have been saved.

In the application of the instrument, I have found that much depends upon a proper lubrication of the silk band and straps. Oil does not answer well, as it renders the silk harsh. A mucillage made by soaking a piece of fine soap in warm water answers better than anything I have yet tried.

That the extractor is superior to the forceps, as a means of applying extractive force in delivery, I am now thoroughly convinced. Its greatest superiority is in the facility and safety with which it can be applied at the superior straight, though I think it fully equal to them under other circumstances.

The only objection that has thus far been urged against the extractor is, that the silk band and straps become saturated with the secretions of the parts, and would be liable to carry any contageous virus from one patient to another, and hence, in time of the prevalence of puerperal fever, might be very mischievous. But a thorough washing with soap and warm water might cleanse it as perfectly as any other fabric; and the probabilities are, that its use would not be called for in the hands of one practitioner more than once during an epidemic of the kind referred to. The suggestion, however, which was made in the *American Journal of Medical Sciences* is entirely worthy of attention, and should put those on their guard who use it, and induce them to observe the utmost care and cleanliness. In case of an epidemic contagion, it might be well to remove the old band and straps after using them and substitute new ones.

